# Psychometric properties of 4-item questionnaire for sleep habits and time in a South American paediatric population

**DOI:** 10.5935/1984-0063.20200048

**Published:** 2021

**Authors:** Marcus Vinicius Nascimento-Ferreira, Augusto César Ferreira de Moraes, Julia Maria Monsalve-Álvarez, Florencia Tello, Keisyanne Araujo-Moura, Carlos A. Delgado, Maria Isabel Bove, Luis A. Moreno, Heraclito Carvalho

**Affiliations:** 1 YCARE (Youth/Child cArdiovascular Risk and Environmental) Research Group, Faculdade de Medicina, Universidade de Sao Paulo, SP, Brazil.; 2 Department of Epidemiology, School of Public Health, University of Sao Paulo, Sao Paulo, SP, Brazil.; 3 University of Antioquia, School of Nutrition and Dietetics - Medellin - Colombia.; 4 Universidad de Buenos Aires, Facultad de Medicina - Buenos Aires - Argentina.; 5 Universidade Federal do Piauí, Biofísica e Fisiologia - Teresina - Piaui - Brazil.; 6 Universidad Nacional Mayor de San. Marcos, Faculty of Medicine - Lima - Peru.; 7 Catholic University of Uruguay, School of Psychology - Montevideo - Uruguay.; 8 Universidad de Zaragoza, Fisiatria y Enfermería - Zaragoza - Spain.

**Keywords:** Child, Adolescent, Surveys and Questionnaires, Reproducibility of Results, Sleep

## Abstract

**Objectives:**

To assess the psychometric properties of 4-item questionnaire about sleep habits and time in South American children (3-10 years) and adolescents (11-18 years).

**Material and Methods:**

We evaluated 459 participants from seven South American cities. Two items from week and weekend days wake up time and bedtime were asked twice, with a 2-week interval. We calculated time spent in bed (subtracting wake up time from bedtime). Participants also answered the Healthy Lifestyle in Europe by Nutrition in Adolescence (HELENA) sleep time questionnaire.

**Results:**

The questionnaire showed acceptable temporal stability in children and adolescents on total days (rho≥0.30; p<0.05). For total days, the questionnaire presented acceptable convergent validity only in children (rho from 0.48 to 0.62; p≤0.01) compared with the HELENA questionnaire.

**Conclusion:**

The 4-item questionnaire is a reliable and valid tool for children; however, its validity is not consistent in adolescents for sleep habits and time.

## INTRODUCTION

Adequate sleep is increasingly recognized as an important determinant of child and adolescent health^1^. However, evaluating sleep habits and time remains a challenge for epidemiological researchers^1^. Regarding subjective instruments, the variability of different applications and interpretations of the questionnaires makes it difficult to standardize a screening instrument in the paediatric population^2^. In addition, there are several factors that can be related to sleep habits and time, such as changes in biological, psychological, cultural, social, and family interactions^1^^,^^3^^,^^4^.

Different multicentre studies from Europe have developed specifically standardized questionnaires for assessing and comparing sleep habits and time^3^^,^^4^. In addition, questionnaires have been mainly developed to evaluate European and North American children and adolescents, while no questionnaires have specifically targeted South American paediatric populations^5^. For this study, we reviewed systematically questionnaires about sleep time^5^. And, we hypothesized that a 4-item questionnaire about sleep habits is sufficient to estimate sleep time (duration) in a South American paediatric population. We adapted the questions from Children’s ChronoType Questionnaire, previously validated^6^^,^^7^.

## MATERIAL AND METHODS

### Design

The current study is part of the South American Youth Cardiovascular and Environmental (SAYCARE) multicentre feasibility study, which collected data from seven South American cities^8^. Data collection was performed in Buenos Aires (Argentina), Lima (Peru), Medellin (Colombia), Montevideo (Uruguay), Santiago (Chile), and São Paulo and Teresina (Brazil). Complete SAYCARE methodology was published previously^8^. Specifically, the reliability of the 4-item questionnaire was assessed through temporal stability (test-retest reliability) and internal consistency. Structural and convergent [compared with the Healthy Lifestyle in Europe by Nutrition in Adolescence (HELENA) questionnaire] validities were also tested. Approval to conduct the study was granted by Brazilian Ethic Committee, under research protocol No. 2,022,542, and also by the other participating centres at the SAYCARE study^9^. The study occurred during the 2015 and 2016 academic years.

### Participants

Regarding sample size estimation, for validity assessment, we used correlation coefficient of 0.40, α=5% and β=10%^5^. For reliability assessment, a subsample was used with a correlation coefficient of 0.5, α=5% and β=10%^7^^,^^10^. The projected sample sizes were 136 participants for the validity study and a subsample of 87 randomly selected participants for the reliability study. To avoid potential sample losses and rejections, we aimed to sample 75% more than was calculated.

From SAYCARE recruited sample (1,067 children and 495 adolescents)^8^, a total of 512 children and adolescents were invited to participate in the 4-item questionnaire validity study. A subsample of 324 participants was included in the reliability study. At the design level, the participants from each centre were equally distributed by sex (male and female) and school type (public and private).

### Data collection

We selected conveniently located schools and sent formal invitations with detailed information about the study. Students who accepted the invitation to participate were required to complete an informed written consent signed by a parent (or legal guardian) and by adolescent participants prior to enrolment. For both the reliability and validity studies, we only considered participants who fully completed all survey sections, including: i) 4-item questionnaire and HELENA (sleep time) questionnaire; ii) birth date; and iii) sex. Participants completed the first (Q1, + HELENA questionnaire) and second (Q2) 4-item questionnaires at home twice with a 2-week interval. Questionnaires for children were completed by their parents (or legal guardians), whereas adolescent participants completed the questionnaires on their own. The age range for children (3-10 years) and adolescents (11-18 years) was based on the World Health Organization criteria^11^.

### Sleep habits and time questionnaire

The 4-item questionnaire about sleep habits and time was adapted from Children’s ChronoType Questionnaire^6^, previously validated [against accelerometer (at least, rho of 0.30 for bedtime)]^7^. The questionnaire addressed sleep habits from the previous week, which were stratified by weekdays and weekend days. A total of four items were assessed, including questions concerning wake up time (“During weekdays/weekend days, what time do you usually wake up?”), bedtime (“During weekdays/weekend days, what time do you usually go to bed?”)^6^. Moreover, we calculated the time spent in bed (by equation: bedtime - wake up time). Additionally, we assessed variables for total days (complete week), the duration (for time spent in bed) and clock time (for wake up time and bedtime) using the following equation^12^:

$$\frac{\left(\mathrm{variable}\;\mathrm{on}\;\mathrm{weekdays}\;x\;5\right)+\left(\mathrm{variable}\;\mathrm{on}\;\mathrm{weekend}\;\mathrm{days}\;x\;2\right)}{7}$$

Demographic and socioeconomic information were also collected.

### HELENA sleep time questionnaire

Information on sleep time (duration) was also collected with the HELENA questionnaire, which has been commonly used in the literature^3^^,^^12^. The HELENA sleep time questionnaire is a 2-item report measure that has demonstrated adequate reliability in multicentre studies of adolescents^3^. Cohen’s weighted kappa showed an almost perfect agreement during weekdays and weekend days (0.81 and 0.96, respectively)^3^. The items assessed were habitual sleep time by the questions “During weekdays, how many hours (and minutes) do you usually sleep?” and “During weekend days, how many hours (and minutes) do you usually sleep?”, a total weekly sleep time was calculated as:

$$\frac{\left(\mathrm{item}\;\mathrm{on}\;\mathrm{weekdays}\;x\;5\right)+\left(\mathrm{item}\;\mathrm{on}\;\mathrm{weekend}\;\mathrm{days}\;x\;2\right)}{7}$$

### Statistical analysis

A *p*-value of <0.05 was considered statistically significant. Stata 14 software (StataCorp, College Station, TX, USA) was used to conduct our analyses. In the sensitivity analyses, differences between categorical variables were estimated using the Chi-square goodness-of-fit test. All items and variables using the Spearman correlation (rho) coefficient for continuous variables assessed the test-retest stability. For internal consistency, Cronbach’s (alpha) coefficient and item-total correlation coefficients were calculated only for items. A value ≥0.30^13^ for rho coefficient and >0.70 for alpha coefficient^14^ were considered acceptable.

Structural validity was assessed with exploratory factor analyses of inter-item polychoric correlations. We applied varimax rotation, considering a factor load index of 0.3 for item exclusion and the eigenvalue-greater-than-one rule (Kaiser’s rule) to decide the number of factors to retain^15^. Convergent validity was assessed by calculating the rho correlation between time spent in bed on the 4-item questionnaire and habitual sleep time on the HELENA.

## RESULTS

For the reliability study, we assessed data from 161 participants (49.7% response rate) and for the validity analysis, we assessed 459 participants (92.7% response rate) (Table 1). Moreover, our sample was composed of 9.3% and 4.1% of the participants from Buenos Aires (Argentina), 23.0% and 19.6% from Lima (Peru), 36.6% and 16.1% from Medellin (Colombia), 6.8% and 9.1% from Montevideo (Uruguay), 5.0% and 10.2% from Santiago (Chile), 17.4% and 16.6% from São Paulo (Brazil), and 1.9% and 24.2% from Teresina (Brazil) for both the reliability and validity studies, respectively.

**Table 1:** Study sample characteristics.

**Children**	**Q1 (N=237)**	**Q2 (N=55)**	**BMI data (N=216)**	**P1**	**P2**
	**%**	**%**	**%**		
**Sex**					
Male	53.3	57.9	52.5	0.10	0.11
Female	46.7	42.1	47.5		
**Age**					
3-5 years	58.7	39.0	57.5	<0.01	0.10
6-10 years	41.3	61.0	42.4		
**Maternal education level**					
Incomplete high school	22.1	23.7	20.4	0.99	0.12
High school	14.7	15.8	15.9		
Technical education	10.2	10.5	10.2		
University degree	52.8	50.0	53.4		
**School type**					
Public	50.2	21.8	62.2	<0.01	0.12
Private	49.8	78.2	37.8		
BMI (median, 25th-75th percentile)			16.6 (15.4-19.1)		
**Adolescents**	**Q1 (N=258)**	**Q2 (N=106)**	**BMI data (N=243)**	**P1**	**P2**
	**%**	**%**	**%**		
**Sex**					
Male	50.0	39.4	48.5	0.64	0.99
Female	50.0	60.6	51.5		
**Age**					
11-14 years	51.9	46.2	52.8	0.11	0.98
15-18 years	48.1	53.8	47.2		
**Maternal education level**					
Incomplete high school	22.6	15.8	22.2	0.42	0.99
High school	25.0	23.7	25.5		
Techinical education	12.3	15.8	12.3		
University degree	40.1	44.7	40.1		
**School type**					
Public	52.7	37.2	48.6	<0.01	0.95
Private	47.3	62.8	51.4		
BMI (median, 25th-75th percentile)			21.1 (19.2-23.5)		

Significant value was set in p < 0.05.; BMI: Body mass index; Q1: Questionnaire first application; Q2: Questionnaire second application; P1: Proportion comparisons between Q1 and Q2 sample distributions; P2: Proportion comparisons between Q1 and participants with BMI data sample distributions.

The test-retest (temporal stability) findings showed acceptable reliability for all items in children and adolescents on total days. However, the internal consistency for weekdays (Cronbach’s alpha of 0.63) in children was not acceptable (Table 2). Exploratory factor analysis revealed 3-factor [labelled as habits on weekend days (1), habits on weekdays (2), and wake up habits(3)] with an explained variance of 89.5% and 2-factor [labelled as habits on weekend days (1) and habits on weekdays (2)] with an explained variance of 71.0% for children and adolescents, respectively. After factor loading and communality analysis, no items have been deleted (Table 3). In addition, 4-item questionnaire showed acceptable convergent validity in children [rho ranging from 0.48 (*p*≤0,01) to 0.60 (*p*≤0,01)] compared with the HELENA sleep time questionnaire; whereas, in adolescents, the convergent validity was not acceptable (rho≤0.20) (Table 4).

**Table 2:** Reliability analysis of the 4-item questionnaire.

Items and variables in children	rho (N=55)	alpha (N=237)
Wake up time on weekdays	0.34**	0.68
Bedtime on weekdays	0.35**	0.60
Time spent in bed^a^ on weekdays	0.09	0.49
All items		0.63
Wake up time on weekend days	0.30**	0.98
Bedtime on weekend days	0.18	0.38
Time spent in bed^a^ on weekend days	0.08	0.21
All items		0.79
Wake up time on total days	0.37**	
Bedtime on total days	0.33**	
Time spent in bed^a^ on total days	0.35*	
**Items and variables in adolescents**	**rho (N=106)**	**alpha (N=258)**
Wake up time on weekdays	0.56**	0.96
Bedtime on week days	0.54**	0.59
Time spent in bed^a^ on weekdays	0.19	0.08
All items		0.72
Wake up time on weekend days	0.92**	0.99
Bedtime on weekend days	0.38**	0.25
Time spent in bed^a^ on weekend days	0.24	0.18
All items		0.79
Wake up time on total days	0.87**	
Bedtime on total days	0.41**	
Time spent in bed^a^ on total days	0.74**	

Moderate (or above) value of spearman correlation was set in rho≥0.30; alpha: Cronbach-alpha coefficient; rho: Spearman correlation coefficient; ^a^: Based on the equation: (bedtime in clock time) - (wake up in clock time);

* *p<*0.05;

** *p*<0.001.

**Table 3:** Exploratory factor analysis of the 4-item questionnaire.

Items in children (N=237)	Factor 1	Factor 2	Factor 3	Uniqueness	Communality (1-uniqueness) %
Wake up time on weekdays			0.8969	0.1547	84.5%
Bedtime on weekdays		0.9590		0.0358	96.4%
Time spent in bed^a^ on weekdays		0.9747		0.0061	99.4%
Wake up time on weekend days	-0.5989		0.5494	0.3073	69.3%
Bedtime on weekend days	0.8997			0.0998	90.0%
Time spent in beda on weekend days	0.9662			0.0276	97.2%
Eigenvalue (proportion of variance)	2.18 (0.36)	2.02 (0.34)	1.16 (0.19)		
Explained variance^b^		0.895 or 89.5%			
**Items in adolescents (N=258)**	**Factor 1**	**Factor 2**		**Uniqueness**	**Communality (1-uniqueness) %**
Wake up time on weekdays				0.9023	10.0%
Bedtime on weekdays		0.9936		0.0050	99.5%
Time spent in bed^a^ on weekdays		0.9693		0.0456	95.4%
Wake up time on weekend days	-0.6211			0.6135	38.6%
Bedtime on weekend days	0.9260			0.1242	87.6%
Time spent in bed^a^ on weekend days	0.9676			0.0485	91.5%
Eigenvalue (proportion of variance)	2.53 (0.42)	1.73 (0.29)			
Explained variance^c^		0.710 or 71.0%			

SAYCARE: South American Youth Cardiovascular and Environmental study;

a Based on the equation: (bedtime in clock time) - (wake up in clock time);

b Proportion and explained variance for the first 3 factors (factor 1, factor 2 & factor 3) identified by using eigenvalue greater than one rule (Kaiser's rule);

c Proportion and explained variance for the first 2 factors (factor 1 & factor 2) identified by using eigenvalue greater than one rule (Kaiser's rule).

**Table 4:** Convergent validity analysis, correlation between 4-item and HELENA questionnaires.

Items in children (N=237)	rho
(SAYCARE) Time spent in bed^a^ on weekdays vs (HELENA) Habitual sleep time on weekdays	0.62**
(SAYCARE) Time spent in bed^a^ on weekend days vs (HELENA) Habitual sleep time on weekend days	0.48**
(SAYCARE) Time spent in bed^a^ on total days vs (HELENA) Habitual sleep time on total days	0.50**
**Items in adolescents (N=258)**	**rho**
(SAYCARE) Time spent in bed^a^ on weekdays vs (HELENA) Habitual sleep time on weekdays	-0.28
(SAYCARE) Time spent in bed^a^ on weekend days vs (HELENA) Habitual sleep time on weekend days	-0.12
(SAYCARE) Time spent in bed^a^ on total days vs (HELENA) Habitual sleep time on total days	0.20

Moderate (or above) values of spearman correlation was set in rho≥0.30; HELENA: Healthy Lifestyle in Europe by Nutrition in Adolescence study; rho: Spearman correlation coefficient; SAYCARE: South American Youth Cardiovascular and Environmental study;

a Based on the equation: (bedtime in clock time) - (wake up in clock time);

* *p <*0.05;

** *p*≤0.01.

## DISCUSSION

As far as we are aware, this is the first valid sleep time and habits questionnaire for a South American paediatric population^5^. In Brazil, the most used questionnaire was not validated, just adapted for Brazilian Portuguese^2^. The 4- item questionnaire showed acceptable reliable and valid for children. In this sense, the questionnaire represents an easy and cost-effective way to measure sleep time and habits in school-aged children.

Regarding reliability, the test-retest stability of our questionnaire was similar to that reported in a recent systematic review, which reported correlations ranging from 0.62 to 0.90 for sleep time duration (including time spent in bed)^5^. An extensive European multicentre study also found that questions used to estimate usual sleep time were reliable^3^. Similarly, studies conducted for sleep habits and time with North American and Asian paediatric populations showed acceptable reliability^6^^,^^7^. Additionally, we found acceptable internal consistency in children and adolescents. These findings are in line with previous sleep questionnaire study statistics reported in children (α=0.76) and adolescents (α=0.74)^6^.

Our findings showed acceptable convergent and predictive validity for assessing sleep habits and time in children. Based on a comprehensive systematic addressing sleep time questionnaire validity, we identified items with high correspondence to the objective measures wake up time and bedtime^5^. In addition, we calculated the time spent in bed (defined as the difference between bedtime and wake up time), which is a variable similar to *assumed sleep duration*^16^. The four items supported six variables for week and weekend days (Table 2), and the structural validity revealed that all of them highly related for habits and time. Our results are in line with the hypothesis that short questionnaires (including fewer domains) can reach better reliability for sleep habits than longer ones^17^.

The present study has several limitations. Although the population sample was robust in size and diversity, participant locations were not equally distributed across the sampled cities for the reliability study. In the reliability study, there was a low response rate for Q2, which we attribute to decreased participant motivation to complete a second (SAYCARE) questionnaire within a short lead-time. However, in post hoc analysis, the sample size from children and adolescents (N=161) remained significant in power (β=6%). Moreover, our sample was selected by convenience, because it was not realistic to include a random and representative sample of South American children and adolescents for our study design. Our questionnaire was not able to assess other sleep time variables (e.g., sleep onset latency, sleep onset time, sleep offset time, and time of nap)^5^ related to sleep disturbances and health outcomes. In addition, we have reports on convergent and predictive validity only in two items (time spent in bed on week and weekend days) and one variable (time spent in bed on total days). And, finally, we have no information about the association between cultural differences and questionnaire psychometric properties, suggesting that the reliability and validity evaluated in this study can be limited to multicentre measurements.

## CONCLUSION

The 4-item questionnaire has acceptable psychometric properties for South American children, constituting a reliable and valid tool for assessing sleep habits and time. In adolescents, the questionnaire shows acceptable reliability and structural validity, but not convergent and predictive validity. This questionnaire gathers sufficient psychometric properties to be tested with an objective tool.

## Appendix

**Supplementary Table 1:** Sample composition for the reliability and validity studies.

Research centers	Argentina	Brazil		Chile	Colombia	Peru	Uruguay	Total
	Buenos Aires	Teresina	São Paulo	Santiago	Medellin	Lima	Montevideo	
**Children**								
Reliability analysis	n=5	n=3	n=11	n=5	n=15	n=8	n=8	55
Validity analysis	n=10	n=75	n=33	n=14	n=35	n=27	n=22	216
**Adolescents**								
Reliability analysis	n=10		n=17	n=3	n=44	n=29	n=3	106
Validity analysis	n=9	n=36	n=43	n=33	n=39	n=63	n=20	243
**Total**								
Reliability analysis	n=15	n=3	n=28	n=8	n=59	n=37	n=11	161
Validity analysis	n=19	n=111	n=76	n=47	n=74	n=90	n=42	459
